# Validation of a stroke model in rat compatible with rt-PA-induced thrombolysis: new hope for successful translation to the clinic

**DOI:** 10.1038/s41598-020-69081-0

**Published:** 2020-07-22

**Authors:** Kajsa Arkelius, Denis Vivien, Cyrille Orset, Saema Ansar

**Affiliations:** 1 Applied Neurovascular Research, Neurosurgery, Department of Clinical Sciences Lund, Faculty of Medicine, Lund University, Klinikgatan 28, BMC C12, 222 42 Lund, Sweden; 2 INSERM UMR-S U1237, Physiopathology and Imaging of Neurological Disorders, GIP Cyceron, Institut Blood and Brain @ Caen-Normandie (BB@C), Bd H. Becquerel, BP 5229, 14074 Caen, France; 3 Department of Clinical Research, Caen-Normandie University Hospital, CHU, 14000 Caen, France

**Keywords:** Animal disease models, Cardiovascular models, Neurological models, Magnetic resonance imaging, Neuroscience, Cell death in the nervous system, Cardiovascular diseases, Thrombosis

## Abstract

The recent clinical trial (DAWN) suggests that recanalization treatment may be beneficial up to 24 h after stroke onset, thus re-opening avenues for development of new therapeutic strategies. Unfortunately, there is a continuous failure of drugs in clinical trials and one of the major reasons proposed for this translational roadblock is the animal models. Therefore, the purpose of this study was to validate a new thromboembolic stroke rat model that mimics the human pathology, and that can be used for evaluating new strategies to save the brain in conditions compatible with recanalization. Stroke was induced by injection of thrombin into the middle cerebral artery. Recombinant tissue-type plasminogen activator (rt-PA) or saline was administrated at 1 h/4 h after stroke onset, and outcome was evaluated after 24 h. Induced ischemia resulted in reproducible cortical brain injuries causing a decrease in neurological function 24 h after stroke onset. Early rt-PA treatment resulted in recanalization, reduced infarct size and improved neurological functions, while late rt-PA treatment showed no beneficial effects and caused hemorrhagic transformation in 25% of the rats. This validated and established model’s resemblance to human ischemic stroke and high translational potential, makes it an important tool in the development of new therapeutic strategies for stroke.

## Introduction

Stroke continues to be one of the leading causes of death and disability worldwide, costing the world billions of EUR each year^1^. In 2016 over 13.6 million people suffered from a stroke, and in over 80% of the cases the stroke was ischemic^2,3^. The only proven drug treatment for acute ischemic stroke is thrombolysis induced by the drug recombinant tissue-type plasminogen activator (rt-PA), either alone or combined with the removal of the thrombi/emboli by thrombectomy. Considering that less than 15% of patients received rt-PA (20–40% of efficacy) and that only 5–10% of patients are eligible for the combined treatment (70–80% of efficacy), there is still a need to develop new treatment strategies^4–8^. In addition to the low numbers of patients treated and low efficacy rates, thrombolysis is also associated with an increased risk of a hemorrhagic transformation, leading to worsen outcomes and increased mortality^9,10^.

Many attempts have been made to discover new treatment options. However, even if the majority of therapies showed beneficial results in preclinical studies, none have shown to improve stroke outcomes in humans^11^. The “bench-to-bedside” stalemate has been heavily discussed, and one of the recurrent reasons behind this is the experimental stroke models used. Even if it is impossible to mimic human stroke in animals completely, the translational potential will increase significantly by enhancing the resemblance between the preclinical and clinical settings.

Since over 80% of all human strokes are thromboembolic, a step to better mimic human stroke in animals is the development of embolic stroke models^12^. Embolic models have been used for decades and have been refined over the years to enable research with thrombolytic drugs which are the only successful therapy in phase III clinical trial^13–15^. The embolic models are based on the usage of pre-formed blood clots. The formation of the clots has developed throughout the years and represent the most significant difference between the embolic models. These pre-formed clots are injected into the cerebral circulation through a catheter placed within the internal carotid artery (ICA). After injection, the clots lodge themselves within the cerebral circulation and disrupt the cerebral blood flow (CBF) in that area. The clots primarily locate themselves within the middle cerebral artery (MCA), however, sometimes the clots travel further in the circulation and secondary occlusions occur. The variances in where the clots are located reduce the reproducibility of the results. In addition, the embolic models have also shown a high mortality rate limiting the usage of existing embolic stroke models^16–21^. Orset et al. overcame this obstacle by successfully establishing a new thromboembolic stroke model in mice by directly injecting thrombin into the MCA using micropipettes. The injection causes a local clot formation in the MCA bifurcation, which prevents CBF within the MCA region. While previous embolic models have shown variability in clot placements, the clot formed through Orset’s model does not move and remains in its original location, generating high reproducibility and low mortality. The model also mimics human ischemic stroke closely by its replication of the thrombi/emboli that gives rise to the blood vessel occlusion in humans; its close clinical resemblance is one of the reasons the model is used by several research groups today^22–26^. With this model’s advantages, translation of the model from one species to another would increase the research possibilities by utilizing the benefits from the different species. Before moving to clinical trials, it is of significant importance to use more than one species as well as performing functional studies. In the rat model, there are several well-established neurological outcome tests^27–29^. The validation of the thromboembolic model in rats will enable us to obtain an improved translation of discoveries from animals to humans.

Therefore, the aim of this study was to validate the thromboembolic stroke model in rats which closely resembles the human ischemic stroke with a low mortality rate and high reproducibility. For additional assessment of the model and its clinical relevance, the effects of both early and late treatment of rt-PA were evaluated.

## Results

### Study outline

**Figure 1:**
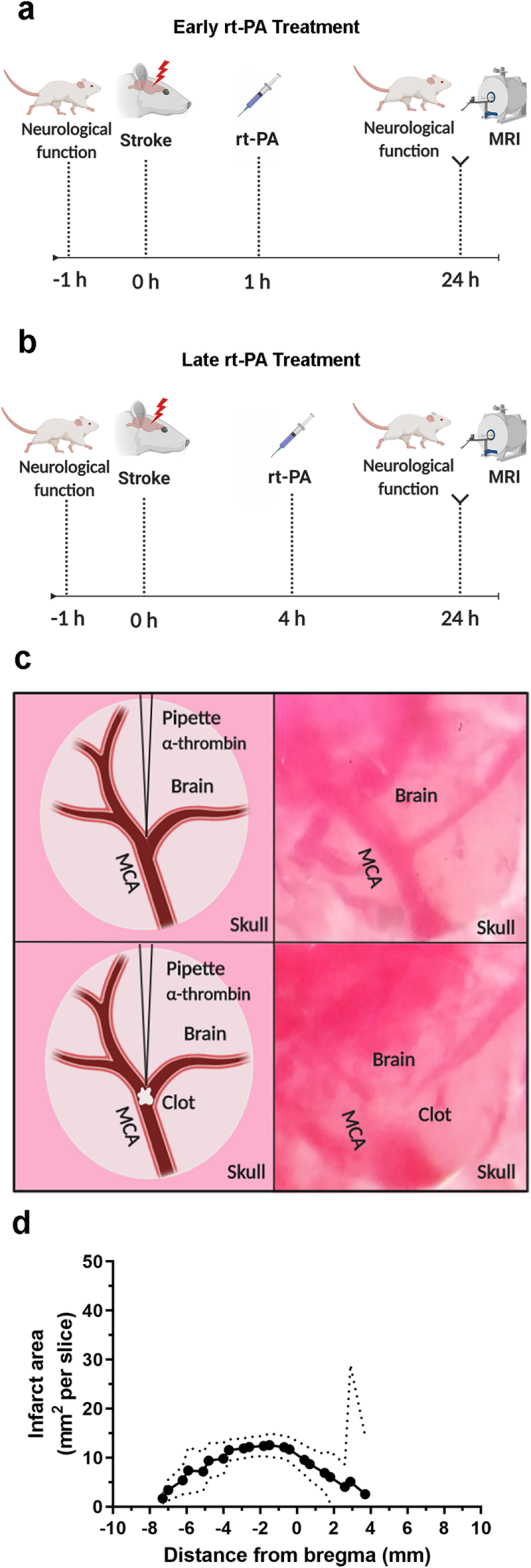
Injection of thrombin into the MCA causes reproducible infarct lesions. (**a**) Illustration of the study outline for early treatment of animals. (**b**) Illustration of the study outline for late treatment of animals. (**c**) Illustration (left column) and visualization (right column) of the in vivo setting after the craniotomy. In the thromboembolic model, the pipette filled with α-thrombin is inserted into the lumen of the MCA bifurcation following thrombin injection (lower row), a formed clot can be visualized within the MCA bifurcation. (**d**) Infarct lesion was reproducible in both size and location, 87.29 (62.97, 111.6) mm^3^, starting at 2.91 (2.15, 3.68) mm and ended at − 6.20 (− 7.51, − 4.89) mm in the cerebral cortex (bregma = 0 mm, n = 7). Data is presented as the area of infarct lesion for each section. Data are expressed as mean and error bars as 95% IC.

Stroke induction was followed by intravenous treatment with either rt-PA or saline administrated at 1 h or 4 h after stroke onset. Neurological function was evaluated through a 28-point neuroscore in all animals prior to surgery and 24 h after stroke induction, which was followed by a magnetic resonance imaging (MRI) scan where infarct lesion and hemorrhagic transformation were evaluated (Fig. 1a–b).

In total, 48 rats were included in this study, and there was no significant difference in the body weight between the different groups (Supplementary Fig. S1a). The mortality rate of this study was 2% (1 rat treated with rt-PA at 4 h, died during the MRI scan 24 h after stroke onset).

### Thromboembolic stroke model in rat

#### Thrombin injection caused blood clot formation and decrease of CBF

Stroke was induced in male Wistar rats with a local injection of thrombin into the MCA lumen. When injected into the bloodstream, thrombin converts fibrinogen into fibrin, which causes the formation of a blood clot (Fig. 1c). Thrombin injection caused a rapid decrease of CBF [mean reduction of CBF, 80.72% (77.97, 83.46) %] in the MCA region, and no difference between treatment groups was observed (Supplementary Fig. S1b). The surgery was considered successful when a stable drop of CBF (65%) for the duration of 1 h was achieved (inclusion/exclusion criterions can be found in Supplementary Table S1). In total six rats were excluded from the study for not making the cut-off of 65% (Supplementary Table S2). For confirmation that it is the thrombin that causes the clot formation and that it is not an effect due to the vascular damage which occurs when the pipette is inserted into the vessel. A control group of n = 5 was injected with saline instead of thrombin. None of the animals showed any indication of reduced CBF after saline injection, neurological function remined normal and no infarct lesion formation was observed. (Supplementary Fig. S2). Blood gases remained stable for the duration of the surgery (Supplementary Table S3).

#### Clot formation induced a reproducible infarct lesion and reduction in neurological function

Thrombin induced clot formation and gave rise to infarct lesions within the cerebral cortex. The infarct lesions were located between 2.91 (2.15, 3.68) mm and − 6.20 (− 7.51, − 4.89) mm in the cerebral cortex (bregma = 0 mm) (Fig. 1d) and induced a decrease in neurological function 24 h after stroke.

### Effects of rt-PA treatment

#### Early rt-PA treatment elicits recanalization the MCA and reduces the infarct lesion

**Figure 2:**
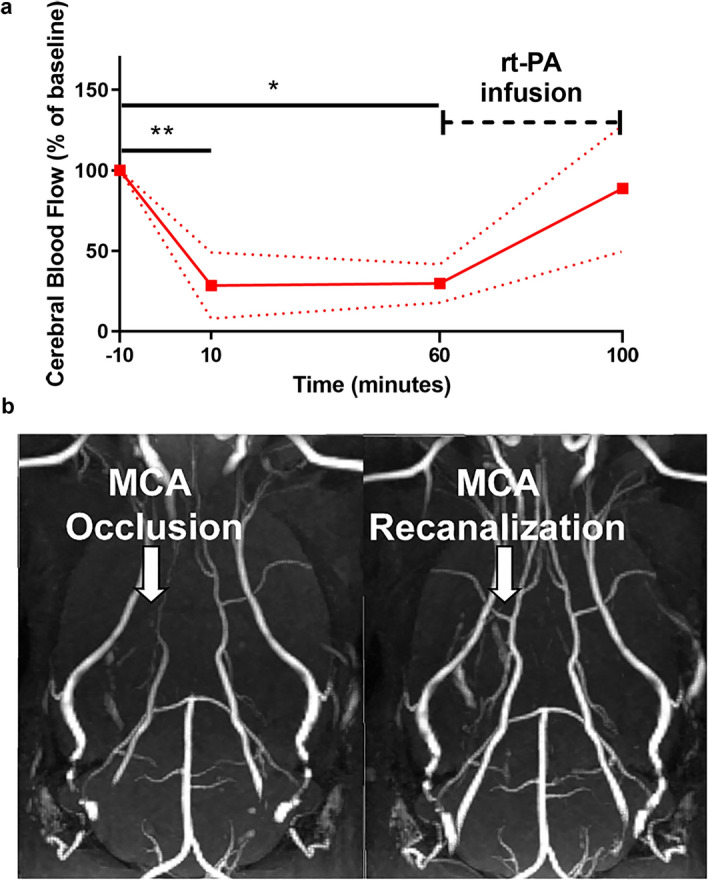
Early treatment with rt-PA recanalizes and restores the blood flow within the MCA. (**a**) CBF was measured in the MCA region throughout the surgery in rats treated with rt-PA 1 h after stroke induction. After thrombin injection at 0 min, a rapid CBF drop can be seen within the MCA region, baseline = 100%. The drop remained stable for 1 h, however, the CBF starts to recover after rt-PA treatment (3 mg/kg) begins. Data are expressed as mean and error bars as 95% IC. (**b**) Treatment with rt-PA 1 h after stroke onset recanalizes the MCA, which is visualized through representative angiographic images, n = 1. The left image is taken 40 min after thrombin injection. The arrow is indicating the location of the MCA, which appears “invisible” due to the lack of blood flow through the vessel. The right image is taken 15 min after ended rt-PA treatment, and here the arrow points to the recanalized MCA.

**Figure 3:**
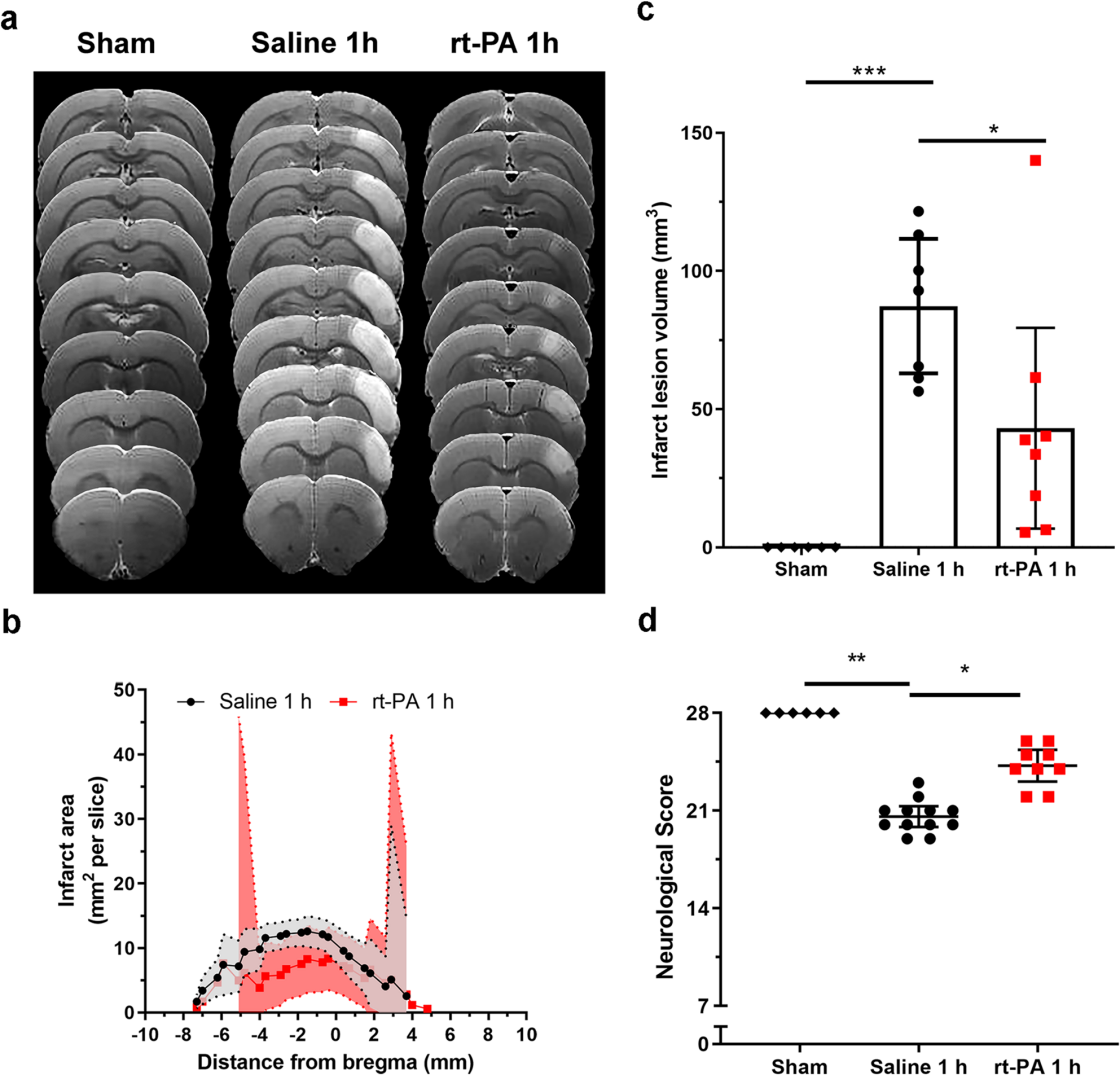
Early treatment with rt-PA improves outcome 24 h after stroke induction. (**a**) Representative T_2_-weighted image visualizing the infarct lesion within the cerebral cortex of sham operated animals, saline, and early rt-PA treated rats. (**b**) In the rt-PA treated animals the infarct was located between 2.33 (1.15, 3.50) mm and − 4.00 (− 5.55, − 2.45) mm, and in saline treated animals between 2.91 (2.15, 3.68) mm and − 6.20 (− 7.51, − 4.89) mm (bregma = 0 mm), both within the cerebral cortex. Data are expressed as mean and error bars as 95% IC. (**c**) Animals treated with rt-PA 1 h after stroke onset had an infarct lesion of 43.10 (6.80, 79.40) mm^3^ and was significantly lower than saline treated animals 87.29 (62.97, 111.6) mm^3^, p = 0.0439. Data are expressed as mean and error bars as 95% IC. (**d**) Neurological function was evaluated 24 h after stroke onset using a 28-point neuroscore. Stroke induced animals treated with saline 1 h (n = 12) after stroke onset showed a significant reduction in neurological function [20.58 (19.84, 21.32)], compared to sham operated animals which showed no reduction in neurological function [28.0 (28.0, 28.0) (n = 6), p = 0.0013]. Treatment with rt-PA (n = 9) resulted in improved neurological function compared to saline [24.22 (23.08, 25.36), p = 0.0346]. Data are expressed as mean and error bars as 95% IC.

Thrombolysis with rt-PA 1 h after stroke caused recanalization of the MCA and reperfusion of CBF in the MCA region (Fig. 2a,b). All stroke-induced animals showed an infarct lesion within the cerebral cortex, while sham operated animals showed no indication of infarct lesion formation. The recanalization of the vessel in rt-PA treated animals prevented the expansion of the infarct core which was located in the cerebral cortex between 2.33 (1.15, 3.50) mm and − 4.00 (− 5.55, − 2.45) mm (bregma = 0 mm) (Fig. 3a,b, Supplementary Fig. S3a). There was a significant reduction in infarct lesion in early rt-PA treated animals 43.10 (6.80, 79.40) mm^3^, (n = 8) compared to the control group 87.29 (62.97, 111.6) mm^3^ (n = 7) p = 0.0439 (Fig. 3c), even if control animals showed an increase in CBF (Supplementary Fig. S4). Differences in infarct lesion volume reflected on the corresponding impact of the stroke on the neurological function. There was a significant neurological impairment in stroke induced animals treated with saline (n = 12) [20.58 (19.84, 21.32)], compared to sham-operated animals (n = 6) that demonstrated no deficit in neurological function [28.0 (28.0, 28.0)], p = 0.0013. The rt-PA treatment (n = 9), with its recanalization and reduction in infarct lesion volume, significantly improved the neurological deficits 24.22 (23.08, 25.36), p = 0.0346 (Fig. 3d), compared to saline treatment.

#### Late rt-PA therapy has no effect on stroke outcome

**Figure 4:**
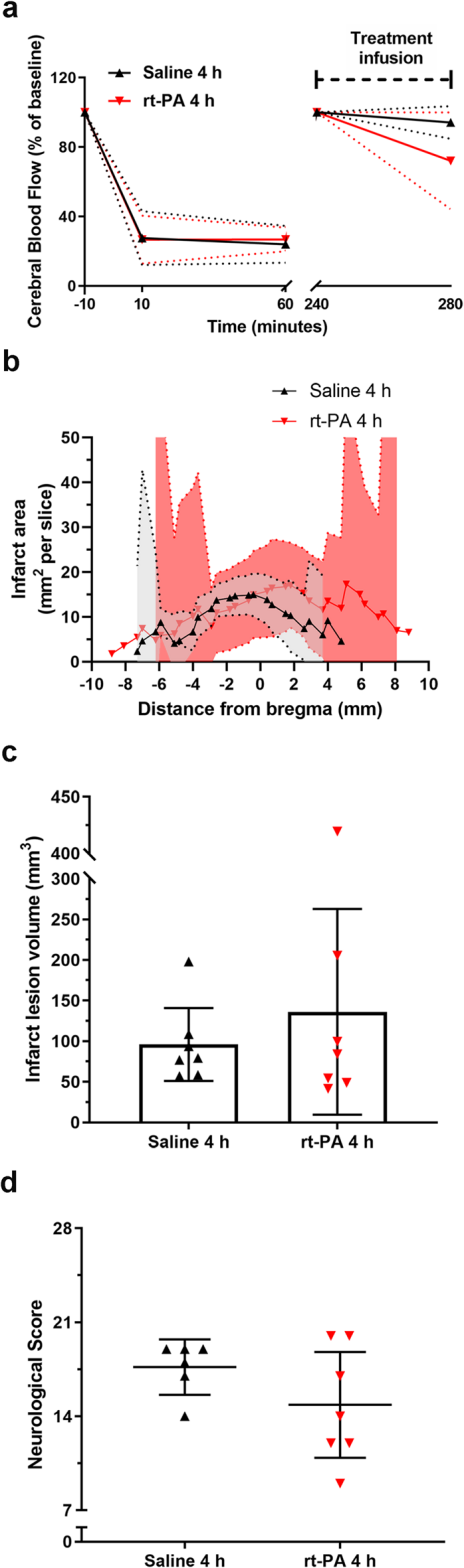
Late rt-PA treatment shows no beneficial effects on stroke outcomes. (**a**) CBF was measured in the MCA region throughout the surgery using a laser Doppler, with a gap between 60–240 min, where the animal was awakened and then re-sedated. Baseline levels measured at − 10 min and then at 240 min after re-sedation was set as 100%. A rapid drop of CBF can be seen after thrombin injection into the MCA, which remained stable for 60 min. Treatment (saline-black or rt-PA-red) was administrated 4 h after thrombin injection, and the CBF remained stable for both treatments. Data are expressed as mean and error bars as 95% IC. (**b**) Treatment with either rt-PA or saline caused infarct lesions located between 3.88 (1.42, 6.32) and − 6.02 (− 8.79, − 3.25) mm, and 3.07 (2.08, 4.06) and − 5.41 (− 6.69, − 4.14) mm (with bregma as 0 mm) respectively, and both within the cerebral cortex. Data are expressed as mean and error bars as 95% IC. (**c**) No difference was observed between either infarct size [saline 96.01 (51.15, 140.9) mm^3^ n = 7, rt-PA 136.2 (9.62, 262.8) mm^3^ n = 7, p = 0.821]. Data are expressed as mean and error bars as 95% IC. (**d**) or in neurological function [saline 17.67 (15.60, 19.73) n = 6, rt-PA 14.86 (10.92, 18.80) n = 7, p = 0.814] between animals treated with either rt-PA or saline 4 h after stroke onset. Data are expressed as mean and error bars as 95% IC.

Treatment with rt-PA at 4 h after stroke onset showed no effect in restoring the CBF (Fig. 4a). The lack of reperfusion lead to no difference in infarct lesion volume between rt-PA treated animals [136.2 (9.62, 262.8) mm^3^ n = 7] and saline treated animals [96.01 (51.15, 140.9) mm^3^ n = 7, p = 0.857] (Supplementary Fig. S3b). Neurological function deficits were also similar between the two groups [rt-PA 14.86 (10.92, 18.80) n = 7, saline 17.67 (15.60, 19.73) n = 6, p = 0.814] (Fig. 4b–d).

#### Late rt-PA therapy increases the risk of hemorrhagic transformation

**Figure 5:**
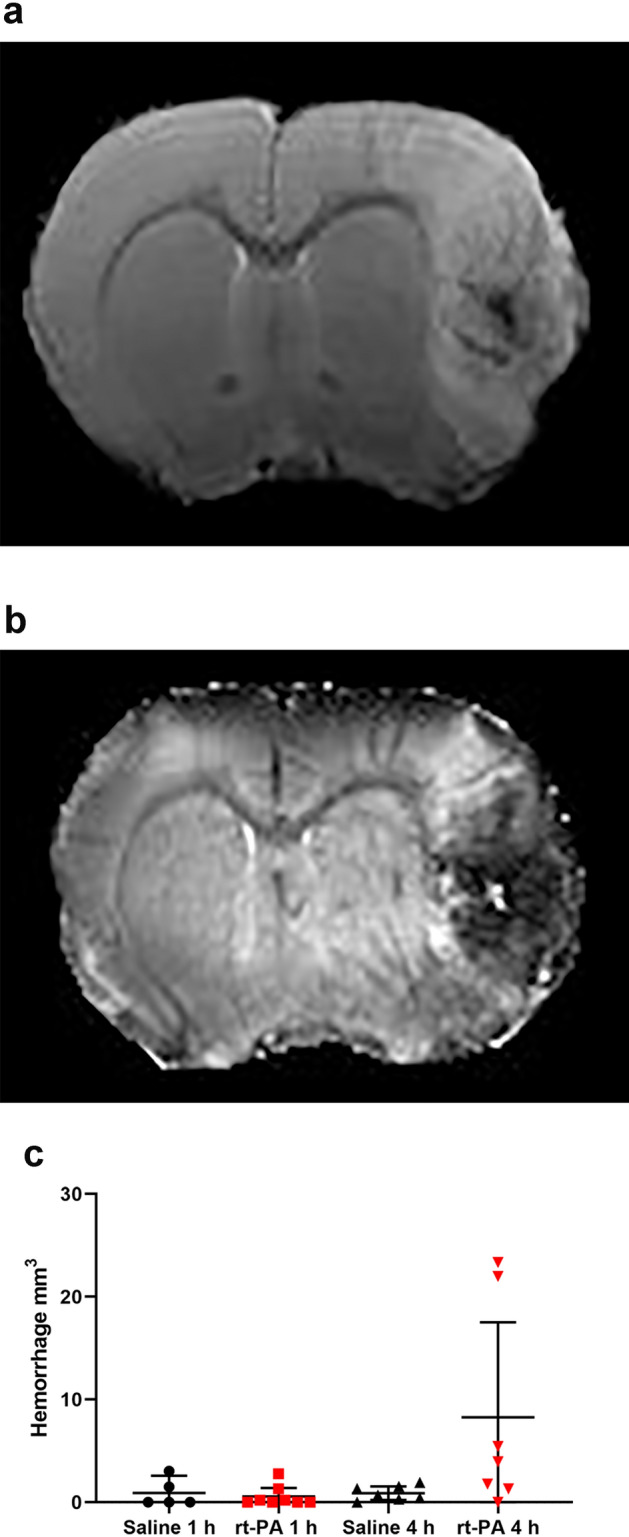
The risk of hemorrhagic transformation increases after late treatment with rt-PA. (**a**) Representative T_2_-weighted (**b**) and T_2_*-weighted images of rat treated with rt-PA 4 h after stroke onset that suffered from a hemorrhagic transformation of the stroke. (**c**) While early rt-PA treatment at 1 h after stroke onset did not induce hemorrhagic transformation, delayed treatment at 4 h resulted in a hemorrhagic transformation in 25% of the animals. Data are expressed as mean and error bars as 95% IC.

While early t-PA treatment at 1 h after stroke onset did not induce hemorrhagic transformation, delayed treatment at 4 h resulted in a hemorrhagic transformation in 25% of the animals (Fig. 5a–c). In these animals, the neurological function was remarkably worsened compared to saline treated animals.

## Discussion

Poor translational possibilities in stroke research imply a lack of transferability between preclinical and clinical results^11^. One contributing factor may be, that the animal models used don´t sufficiently represent the pathophysiology naturally occurring stroke^13^. Thromboembolic stroke models mimic human stroke more closely than other models since they imitate the cause of the occlusion, which in most human cases is via thromboembolism^30^. In addition, they are the best models to study thrombolytic strategies due to the models’ use of thromboembolisms, which is the only beneficial drug treatment in phase III clinical trials. However, previous thromboembolic models have accomplished limited significance in the stroke research field due to their variability in producing accurate and reproducible infarct volumes often accompanied with too high mortality rates. Thus, there remains a need to develop appropriate animal models for preclinical stroke research to allow for a better translation of discoveries from animals to humans. Orset et al. developed a novel thromboembolic model in mice that demonstrated lower mortality and better reproducibility compared to previous thromboembolic models^22^. In the current study, we transferred and validated the same model in rats. It is of significant importance to study the functional outcome after treatment strategies in stroke research and neurological outcome tests are well-established in rats. New treatments strategies for stroke needs to be developed and with the unmet need in the development of new treatment strategies for stroke, the thromboembolic model presented in this study will play a vital role in the evaluation of new strategies in preclinical stroke research.

This thromboembolic stroke model utilizes the properties of thrombin to cause a local clot formation in the MCA^31^. This mimic the human ischemic stroke where a thrombi/embolus occlusion in the MCA is the most common site of occlusion in patients diagnosed with ischemic stroke^32–34^. As in humans, the local clot formation causes a rapid decrease in CBF in the MCA region of the brain, starting the pathophysiology of ischemic stroke. The infarct lesion formed was located in the cerebral cortex and showed reproducibility in its location and its ability to reduce in neurological function. This is also obtained in mice but has been lacking in previous embolic models^14^. Previous embolic models used preformed homologous/autologous clot/clots injected into the MCA through a catheter placed in the ICA^15,19,20^. These embolic models have shown high mortality rates and variability in clot placement, infarct lesion size and location^15,35,36^. The variability of the models may very well resemble the clinical setting; however, the variability also makes the usage of the models in the assessment of new therapeutic strategies arguable. The thromboembolic stroke model requires the performance of a thrombectomy, and while it is more invasive than previous embolic models it has lower mortality rates. High mortality rates can influence the results of studies and is known to be high in previous embolic models where it occurs both within and beyond the 24 h timeframe. In our study, only one animal died whilst under anaesthesia during the MRI scan at 24 h. Analyses showed it suffered from a considerable hemorrhage within the infarct lesion. Previous studies have shown this thromboembolic mouse stroke model to have a low mortality both at 24 h, but also long-term studies which is indicative of its safety^37^. Our new rat model of ischemic stroke likewise displays high reproducibility and low mortality. This mimicked the results from the model in mice, which are underlining its usability in preclinical stroke research for developing new therapeutics.

While cell death and/or infarct lesion are easy to use as outcomes in preclinical studies, the primary outcome in clinical studies is a range of functional outcome assessments of the patients^38^. To close the gap to the clinical research it is of substantial importance to evaluate the sensorimotor function. We revealed a measurable decrease in neurological function after stroke using a well-established sensorimotor neuroscore^27^. The 28-point neuroscore tests a broadness of sensorimotor functions which gave us the ability to detect even small changes in neurological function due to e.g. infarct lesions only affects the sensorimotor cortex. However, before moving forward to the evaluation of new treatment targets, additional neurological tests need to be performed on this animal model.

The rt-PA treatment has several limitations, such as its neurotoxicity through rt-PA’s ability to interact with NDMA receptors and an increased risk of hemorrhagic transformation^30,39^. Treatment with rt-PA is still the only approved drug for acute ischemic stroke. It has the capability to lyse thrombi/emboli and improve stroke outcome^40^. We evaluated the effect of early and late rt-PA in this model. Our study clearly resembled the clinical situation. When rt-PA is given at an early stage^5^, it caused recanalization of the MCA and restored the CBF preventing both the advancement of the infarct core and reduction in neurological function. Spontaneous recovery of the CBF was observed in saline-treated animals at the early time-point, even though there was still a significant difference between infarct lesion volume and neurological function between the saline and rt-PA treatment. Clinical studies show that reperfusion is a better predictor of stroke outcome compared to recanalization. This is mostly due to the fact that recanalization doesn´t always lead to reperfusion^41^. This no-reflow phenomenon within the microvasculature may explain why we see a spontaneous recovery of CBF in the MCA region in saline treated animals. Still there was a significantly larger infarct volume compared with rt-PA treated animals. We observed no difference in neurological function or infarct lesion in animals treated with saline with or without reperfusion, indicating that we cannot conclude the stroke outcome from our CBF data measured by Laser Doppler alone. It is crucial to keep in mind that the Laser Doppler measures only change in the territory of the MCA during surgery and that the observed outcome is possibly due to an interplay of factors such as vascular reserve, collaterals, and autoregulation mechanisms. Both clinical and preclinical studies show an indication that the body temperature at administration of rt-PA may influence the results of the rt-PA efficacy^42^. Hyperthermia at administration has shown to worsen the stroke outcome after rt-PA clinical treatment, but it is also seen preclinically that increased temperatures reduces the beneficial effects of rt-PA therapy^42,43^. In our study, body temperature was monitored and kept within the normal physiological parameters thus limiting the influence of body temperature on the rt-PA treatment.

Several studies indicate that treatment with rt-PA increases the risk of hemorrhagic transformation, which worsens the outcome after stroke^9,40^. Therefore, treatment with rt-PA is restricted only to be given within 4.5 h from stroke onset in the clinic^39^. Previous preclinical studies showed that rt-PA treatment at 3 h after stroke onset might worsen stroke outcome and increase the risk of hemorrhagic transformation replicating the clinical situation^24,25,44^. The time-point of 4 h for delayed rt-PA treatment was chosen in order to enable us to move closer to the clinical end-point^40^. In the present study, rt-PA treatment 4 h after stroke onset showed no beneficial effects in neurological function or ischemic lesion volume. In 25% of the animals treated with late rt-PA, major hemorrhagic areas were found, associated with decreased neurological function and an increase in infarct lesion volume. García-Ye´benes et al. evaluated the incidence of hemorrhages in the mouse model and showed that late treatment of rt-PA (3 h after stroke) increased the risk of hemorrhagic transformation and gave rise to parenchymal hemorrhages in over 30% of the mice^25^. The results from the present and previous studies in mice replicates the increased risk of hemorrhagic transformation observed in the clinic when rt-PA treatment is delayed^25,30,39,40^. In several animal studies an increased risk of hemorrhagic transformation has been observed when rt-PA was given 3–6 h after stroke onset^25,45^. In humans a significant increase in hemorrhagic transformation is seen when treatment is given between 5–6 h after stroke onset^46^. Even if the time may not be directly translatable, the results still represent clinical outcomes. The new DAWN trial indicates that recanalization can be beneficial up to 24 h. This proposes that treatment with rt-PA can become of importance even after the time-window of 4.5 h if the risk of hemorrhage can be prevented^47^. This animal model of ischemic stroke, now established in both mice and rats, shows great potential for investigating new strategies to prevent the rt-PA induced hemorrhagic transformation of the stroke.

Dosage determination was based on previous studies and the results of rt-PA usage within the clinic. While the clinic is using a dosage of 0.9 mg/kg, previous preclinical studies have demonstrated discrepancies between different dosage. Early in vitro results comparing rt-PA efficiency on different species displayed that rt-PA in rodents had a tenfold less efficacy than in humans^48^. Based on this study, many preclinical studies have moved on using a dosage tenfold stronger than what is used in the clinic. The higher dosage of 10 mg/kg is used in the thromboembolic stroke model in mice and has shown similar results that are seen in the clinic^22,23,25^. Studies have also produced irregularities in the efficacy of rt-PA when comparing different dosages in in vivo models^49,50^. Dosages of 4.5 mg/kg rt-PA and above have been seen to have too high efficacy of recanalization that is not seen in the clinic, while dosages of 1.8 mg/kg and under has shown too low efficacy^49^. Taking this in consideration we performed a dose–response evaluation of 1 mg/kg, 3 mg/kg and 10 mg/kg. As seen previously, 1 mg/kg showed no beneficial effects in either restoration of CBF, reduced infarct lesion, or improved neurological function. Animals treated with the dosage of 10 mg/kg showed indications of systematic bleedings and were euthanized. However, evaluation of the dosage of 3 mg/kg showed similar results as to be expected in the clinic with restoration of CBF, reduced infarct lesion volume, and improved stroke outcome. Therefore, we continued with a dosage of 3 mg/kg rt-PA within this study.

In conclusion, we validated the thromboembolic stroke model in rat, which closely resembled the ischemic stroke and therapeutic window for rt-PA in humans. A model that clearly shows great translational potential was established and validated. This model can be used to further study the pathophysiology of cerebral ischemia and as an important tool in the search for new treatment strategies, especially thrombolytic therapies either alone or in combination with other strategies.

## Materials and methods

### Thromboembolic stroke

Thromboembolic stroke was induced in male Wistar rats (255–385 g; Janvier, France) with a local injection of α-thrombin directly into the MCA lumen. The model is based on the thromboembolic mouse model previously described by Orset et al.^22^. Prior to surgery, a micropipette (Assistant, Germany) was prepared using a Moving-Coil Microelectrode Puller (Campden Instruments Limited, USA) and loaded with 12 UI human α-thrombin (4 UI/μl, Nordic Diagnostica AB, Sweden). Rats were anesthetized with 3% isoflurane and maintained with 1.5–2% isoflurane (Baxter Medical AB, USA) in N_2_O:O_2_ (70:30). Temperature was monitored through a rectal thermometer and maintained at 37 ± 1 °C using a heating pad. The rat was then placed in a stereotactic device before a vertical incision was made between the eye and ear. The temporal muscle was retracted and thinning of the skull bone localization of the MCA bifurcation was possible. A craniectomy was performed, and the clearing of the dura revealed the MCA bifurcation. The laser Doppler flow probe (AD Instruments, UK) was placed over the MCA region for monitoring the CBF during the duration of the surgery. The pipette was inserted into the MCA lumen through the bifurcation and α-thrombin was then slowly injected (Fig. 1c). To allow the clot to stabilize, the pipette was left in its position for a minimum of 20 min. Blood gases (pO_2_, pCO_2_ and pH) and glucose levels were monitored through a tail artery catheter both pre-and post-stroke. For sham operated animals the pipette was inserted into the MCA, but nothing was injected. Evaluation of any clot formation due to vascular damage from the pipette placing in the MCA, a pipette containing saline was placed into control animals and the saline was injected into the MCA.

### Treatment

Dosage of 3 mg/kg (1 mg/ml) rt-PA (Alteplase, Boehringer Ingelheim AB, Germany) was chosen after evaluation of 1, 3, and 10 mg/kg, where 1 mg/kg showed no improvement and 10 mg/kg caused bleedings (data not shown). Treatment (rt-PA or saline) was randomized and administrated intravenously, starting with a 10% bolus dose followed by a 40 min infusion administrated either at 1 h or 4 h after stroke induction. Animals treated at the late time point were awakened 1 h after stroke induction and then re-anaesthetized for treatment at 4 h.

### Neurological evaluation

Neurological function was assessed by the 28-point neuroscore test, which consists of 11 tasks that are based on the performance of the rat^27^. The animal’s failure in performing a task lowers the score, indicating a decrease in neurological function while a maximum score of 28 points indicates the healthy function of the animal. Neurological function was assessed both before surgeries to gain baseline values and 24 h post-stroke onset. A full score of 28-points before surgery was required for the inclusion of the animal in the study.

### Magnetic resonance imaging

MRI was performed 24 h post-stroke for evaluation of the infarct volume and hemorrhagic transformation. Visualization of blood flow in the MCA before and after rt-PA treatment was performed 40 min after thrombin injection and after early rt-PA treatment.

The anaesthesia was maintained by inhalation of 2% isoflurane in N_2_O:O_2_ (70:30) during the imaging procedure. The breathing rate and body temperature were monitored during the imaging procedure. A 9.4 T preclinical MRI horizontal bore scanner (Biospec 94/20, Bruker, Germany) was used. For transmission a quadrature volume resonator (112/087) coil was used and for reception a rat brain 2 × 2 phased array coil was used (Bruker, Germany).

#### T_2_-weighted imaging

^1^H T_2_-weighted images were acquired using the RARE sequence as follows: 25 axial slices, slice thickness 0.8 mm, 256 × 146 matrix, in plane resolution 137 × 137 μm^2^, TR = 2,700 ms, TE = 33 ms, bandwidth 33 kHz, TA = 2 min 25 s.

#### T_2_^*^-mapping

^1^H T_2_^*^-maps were reconstructed from a multigradient echo sequence acquired with parameters as above except: TR = 1,800 ms, TE = 3.5 ms to 58.5 ms in steps of 5 ms, bandwidth 69 kHz, TA = 3 min 18 s.

#### Time-of-flight angiography

^1^H 3D time-of-flight angiography was acquired as: 200 × 200x160 matrix, resolution 150 × 165 × 150 μm^2^, TR = 10 ms, TE = 1.8 ms, bandwidth 96 kHz, TA = 4 min 24 s.

#### Analysation of magnetic resonance imaging

The T_2_-weighted images were acquired to measure the infarct lesion volume, and T2* images for measurement of the volume of hemorrhagic regions. Images were analysed using ImageJ and the region of interest corresponding to the whole ischemic lesion or hemorrhage was manually delineated by an operator blinded to the treatment groups. The damage was reported as infarct volume/hemorrhage (mm^3^).

### 2,3,5-Triphenyltetrazolium chloride (TTC) staining

After euthanisation, the brains from the control group was dissected and cut in 2 mm coronal slices before they were immersed in 1% of TTC for 20 min in 37 °C.

### Statistical analysis

Data is presented as mean and its 95% confidence interval (CI). P < 0.05 was considered statistically significant and “n” refers to the number of animals. Statistical analyses were performed using GraphPad Prism version 7.02 for Windows, GraphPad Software, USA, www.graphpad.com. Physiological parameters data was analysed using Wilcoxon matched-pairs signed rank text and the changes in CBF was analysed through a nonparametric Friedman’s multiple comparisons test, while the differences of the drop of CBF between treatment groups were analysed using Dunn’s multiple comparisons test. The nonparametric uncorrelated Dunn’s test was used to analyse the difference in infarct volume and 28-point neuroscore between the four treatment groups. Blinding was revoked after analyses were finalized. Based on preliminary data, our power calculation showed a sample size of n = 8 per treatment group to detect a difference in neurological function between groups with a 2-sided 5% significance level and a power of 80%. Power calculation was done with G*power 3.1.9.4.

### Ethics

All procedures and animal experiments were performed in full compliance with the ARRIVE and the European Community Council Directive (2010/63/EU) for Protection of Vertebrate Animals Used for Experimental and other Scientific Purposes guidelines. Ethical permit was approved by the Malmö-Lund Institutional Ethics Committee under the Swedish National Department of Agriculture (Animal Inspectorate License No. M125-16).

## Supplementary information

Supplementary Information 1.

## Data Availability

The data that support the findings of this study are available from the corresponding author upon reasonable request.

## References

[1] Luengo-Fernandez R, Violato M, Candio P, Leal J (2019). Economic burden of stroke across Europe: A population-based cost analysis. Eur. Stroke J..

[2] Collaborators GBDS (2019). Global, regional, and national burden of stroke, 1990–2016: A systematic analysis for the Global Burden of Disease Study 2016. Lancet Neurol..

[3] Chia NH, Leyden JM, Newbury J, Jannes J, Kleinig TJ (2016). Determining the number of ischemic strokes potentially eligible for endovascular thrombectomy: A population-based study. Stroke J. Cereb. Circ..

[4] Bhatia R (2010). Low rates of acute recanalization with intravenous recombinant tissue plasminogen activator in ischemic stroke: Real-world experience and a call for action. Stroke J. Cereb. Circ..

[5] Lee KY (2007). Early recanalization after intravenous administration of recombinant tissue plasminogen activator as assessed by pre- and post-thrombolytic angiography in acute ischemic stroke patients. Stroke J. Cereb. Circ..

[6] Saver JL (2015). Stent-retriever thrombectomy after intravenous t-PA vs. t-PA alone in stroke. N. Engl. J. Med..

[7] McMeekin P (2017). Estimating the number of UK stroke patients eligible for endovascular thrombectomy. Eur. Stroke J..

[8] Tsivgoulis G (2016). Eligibility for mechanical thrombectomy in acute ischemic stroke: A phase IV multi-center screening log registry. J. Neurol. Sci..

[9] Tanne D (2002). Markers of increased risk of intracerebral hemorrhage after intravenous recombinant tissue plasminogen activator therapy for acute ischemic stroke in clinical practice: The Multicenter rt-PA Stroke Survey. Circulation.

[10] National Institute of Neurological, D., Stroke rt, P. A. S. S. G. (1995). Tissue plasminogen activator for acute ischemic stroke. N Engl J Med.

[11] O'Collins VE (2006). 1,026 experimental treatments in acute stroke. Ann. Neurol..

[12] Benjamin EJ (2018). Heart disease and stroke statistics-2018 update: A report from the American Heart Association. Circulation.

[13] Hossmann KA (2012). The two pathophysiologies of focal brain ischemia: Implications for translational stroke research. J. Cereb. Blood Flow Metab..

[14] Kilic E, Hermann DM, Hossmann KA (1998). A reproducible model of thromboembolic stroke in mice. NeuroReport.

[15] Zhang Z (1997). A new rat model of thrombotic focal cerebral ischemia. J. Cereb. Blood Flow Metab..

[16] Rasmussen RS, Overgaard K, Pakola S, Boysen G (2008). Effects of microplasmin on recovery in a rat embolic stroke model. Neurol. Res..

[17] Orset, C., Haelewyn, B., Vivien, K., Vivien, D. & Young, A. R. In *Rodent Models of Stroke* (ed. Ulrich Dirnagl) 55–70 (Humana Press, Totowa, 2010).

[18] Macrae IM (2011). Preclinical stroke research—advantages and disadvantages of the most common rodent models of focal ischaemia. Br. J. Pharmacol..

[19] Kaneko D, Nakamura N, Ogawa T (1985). Cerebral infarction in rats using homologous blood emboli: Development of a new experimental model. Stroke J. Cereb. Circ..

[20] Kudo M, Aoyama A, Ichimori S, Fukunaga N (1982). An animal model of cerebral infarction. Homologous blood clot emboli in rats. Stroke J. Cereb. Circ..

[21] Wang CX, Todd KG, Yang Y, Gordon T, Shuaib A (2001). Patency of cerebral microvessels after focal embolic stroke in the rat. J. Cereb. Blood Flow Metab..

[22] Orset C (2007). Mouse model of in situ thromboembolic stroke and reperfusion. Stroke J. Cereb. Circ..

[23] Orset C (2016). Efficacy of alteplase in a mouse model of acute ischemic stroke: A retrospective pooled analysis. Stroke J. Cereb. Circ..

[24] Campos F (2013). Fingolimod reduces hemorrhagic transformation associated with delayed tissue plasminogen activator treatment in a mouse thromboembolic model. Stroke J. Cereb. Circ..

[25] Garcia-Yebenes I (2011). A mouse model of hemorrhagic transformation by delayed tissue plasminogen activator administration after in situ thromboembolic stroke. Stroke J. Cereb. Circ..

[26] Langhauser FL (2012). Thromboembolic stroke in C57BL/6 mice monitored by 9.4 T MRI using a 1H cryo probe. Exp. Transl. Stroke Med..

[27] Encarnacion A (2011). Long-term behavioral assessment of function in an experimental model for ischemic stroke. J. Neurosci. Methods.

[28] Boltze J (2006). The stairway: A novel behavioral test detecting sensomotoric stroke deficits in rats. Artif. Organs.

[29] Kleim JA, Boychuk JA, Adkins DL (2007). Rat models of upper extremity impairment in stroke. ILAR J..

[30] del Zoppo GJ (1992). Recombinant tissue plasminogen activator in acute thrombotic and embolic stroke. Ann. Neurol..

[31] Di Cera E, Dang QD, Ayala YM (1997). Molecular mechanisms of thrombin function. Cell Mol. Life Sci..

[32] Saqqur M (2007). Site of arterial occlusion identified by transcranial Doppler predicts the response to intravenous thrombolysis for stroke. Stroke J. Cereb. Circ..

[33] Broussalis E (2012). Current therapies in ischemic stroke. Part B. Future candidates in stroke therapy and experimental studies. Drug Discov. Today.

[34] Durukan A, Tatlisumak T (2007). Acute ischemic stroke: Overview of major experimental rodent models, pathophysiology, and therapy of focal cerebral ischemia. Pharmacol. Biochem. Behav..

[35] Rasmussen RS, Overgaard K, Hildebrandt-Eriksen ES, Boysen G (2006). d-Amphetamine improves cognitive deficits and physical therapy promotes fine motor rehabilitation in a rat embolic stroke model. Acta Neurol. Scand..

[36] Overgaard K, Rasmussen RS, Johansen FF (2010). The site of embolization related to infarct size, oedema and clinical outcome in a rat stroke model—further translational stroke research. Exp. Transl. Stroke Med..

[37] Drieu A (2019). Immune responses and anti-inflammatory strategies in a clinically relevant model of thromboembolic ischemic stroke with reperfusion. Transl. Stroke Res..

[38] Roberts L, Counsell C (1998). Assessment of clinical outcomes in acute stroke trials. Stroke J. Cereb. Circ..

[39] Hill MD, Buchan AM, Canadian Alteplase for Stroke Effectiveness Study, I (2005). Thrombolysis for acute ischemic stroke: results of the Canadian Alteplase for Stroke Effectiveness Study. CMAJ.

[40] Hacke W (2008). Thrombolysis with alteplase 3 to 4.5 hours after acute ischemic stroke. N. Engl. J. Med..

[41] Cho TH (2015). Reperfusion within 6 hours outperforms recanalization in predicting penumbra salvage, lesion growth, final infarct, and clinical outcome. Stroke J. Cereb. Circ..

[42] Ueno T (2018). Association of survival and hyperthermia after rt-PA for ischemic stroke. Acta Neurol. Scand..

[43] Noor R, Wang CX, Shuaib A (2005). Hyperthermia masks the neuroprotective effects of tissue plaminogen activator. Stroke J. Cereb. Circ..

[44] Garcia-Culebras A (2017). Toll-like receptor 4 mediates hemorrhagic transformation after delayed tissue plasminogen activator administration in in situ thromboembolic stroke. Stroke J. Cereb. Circ..

[45] Dijkhuizen RM, Asahi M, Wu O, Rosen BR, Lo EH (2001). Delayed rt-PA treatment in a rat embolic stroke model: Diagnosis and prognosis of ischemic injury and hemorrhagic transformation with magnetic resonance imaging. J. Cereb. Blood Flow Metab..

[46] Clark WM, Albers GW, Madden KP, Hamilton S (2000). The rtPA (alteplase) 0- to 6-hour acute stroke trial, part A (A0276g): Results of a double-blind, placebo-controlled, multicenter study. Thromblytic therapy in acute ischemic stroke study investigators. Stroke J. Cereb. Circ..

[47] Nogueira RG (2018). Thrombectomy 6 to 24 hours after stroke with a mismatch between deficit and infarct. N. Engl. J. Med..

[48] Korninger C, Collen D (1981). Studies on the specific fibrinolytic effect of human extrinsic (tissue-type) plasminogen activator in human blood and in various animal species in vitro. Thromb. Haemost..

[49] Tomkins AJ, Hood RJ, Levi CR, Spratt NJ (2015). Tissue Plasminogen Activator for preclinical stroke research: Neither "rat" nor "human" dose mimics clinical recanalization in a carotid occlusion model. Sci. Rep..

[50] Haelewyn B, Risso JJ, Abraini JH (2010). Human recombinant tissue-plasminogen activator (alteplase): Why not use the 'human' dose for stroke studies in rats?. J. Cereb. Blood Flow Metab..

