# Supplementary material for: Validation of a stroke model in rat compatible with rt-PA-induced thrombolysis: new hope for successful translation to the clinic
Source: Sci Rep. 2020 Jul 22;10:12191. doi: 10.1038/s41598-020-69081-0 (PMC7376012; doi:10.1038/s41598-020-69081-0)
Supplement: Supplementary file 1 — Supplementary Information 1. [file 41598_2020_69081_MOESM1_ESM.pdf]

Online Supplement

Validation of a stroke model in rat compatible with rt-PA-induced thrombolysis: new hope for successful translation to the clinic

Kajsa Arkelius[1#], Ms; Denis Vivien[2,3], Ph.D; Cyrille Orset[2*], Ph.D; Saema Ansar[1*], Ph.D

[1]Applied Neurovascular Research, Neurosurgery, Department of Clinical Sciences Lund, Faculty of Medicine, Lund University, Klinikgatan 28, BMC C12, 222 42 Lund, Sweden

[2]INSERM UMR-S U1237, Physiopathology and Imaging of Neurological Disorders, GIP Cyceron, Institut Blood and Brain @ Caen-Normandie (BB@C), Bd H. Becquerel, BP 5229, Caen, F-14074 France

[3]Department of Clinical Research, Caen-Normandie University Hospital, CHU, Caen, F-14000, France

* Saema Ansar and Cyrille Orset contributed equally to this article

#Corresponding author
Kajsa Arkelius Applied Neurovascular Research, Neurosurgery, Department of Clinical Sciences Lund, Faculty of Medicine, Lund University, Klinikgatan 28, BMC C12, 222 42 Lund, Sweden
E-mail: kajsa.arkelius@med.lu.se

Supplemental Tables

Table S1. Inclusion and exclusion criteria.

**Inclusion Criteria**	A full score of 28–points before surgery were required for inclusion of the animal in the study.<br><br>A drop of 65 % of CBF that remained stable for a minimum of 1 h.<br><br>Visual confirmation of clot formation.
**Exclusion criteria**	An infarct volume below 5 mm^3 (exception was sham operated animals)

Table S2. Included / Excluded animals.

**Treatment Group**	**Included**	**Excluded**	**Exclusion criteria**	**Number of animals (n)**
Saline 1 h	Included			12
Saline 1 h		Excluded	CBF drop < 65%	3
rt-PA 1 h	Included			7
rt-PA 1 h		Excluded	Infarct volume < 5 mm^3	1
rt-PA 1 h		Excluded	CBF drop < 65%	1
Saline 4 h	Included		CBF drop < 65%	7
Saline 4 h		Excluded	CBF drop < 65%	1
rt-PA 4 h	Included			7
rt-PA 4 h		Excluded	CBF drop < 65%	1
Sham	Included			6
Control group (Sham injected with saline)	Included			5

Table S3. Physiological parameters.

**Physiological parameter**	**Pre-stroke (n= 6)**	**Post-stroke (n=6)**
pH	7.40 (7.39, 7.72)	7.36 (7.31, 7.41)
pCO2 (kPa)	6.33 (5.61, 7.05)	6.70 (5.72, 7.68)
pO2 (kPa)	16.28 (13.38, 19.19)	14.77 (12.46, 17.08)
Glucose (mmol/L)	14.73 (13.00, 16.47)	13.18 (9.06, 17.31)

Supplemental Figures.

Figure S1

a

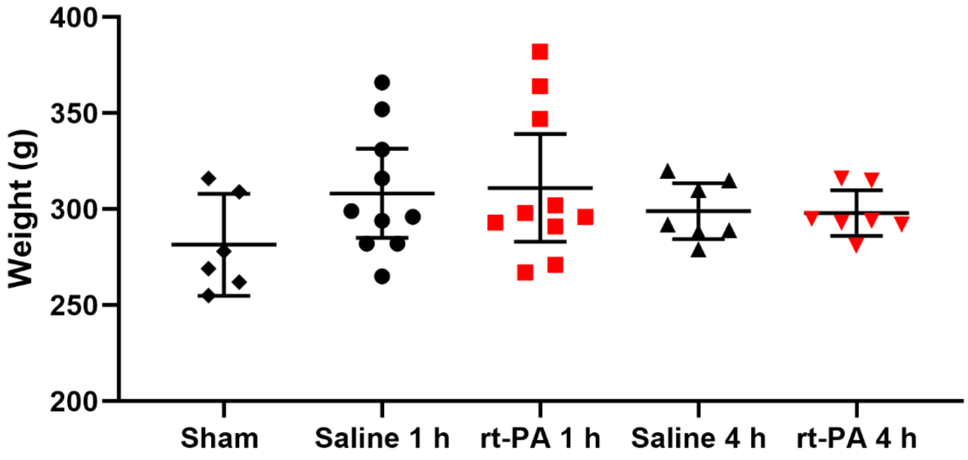

b

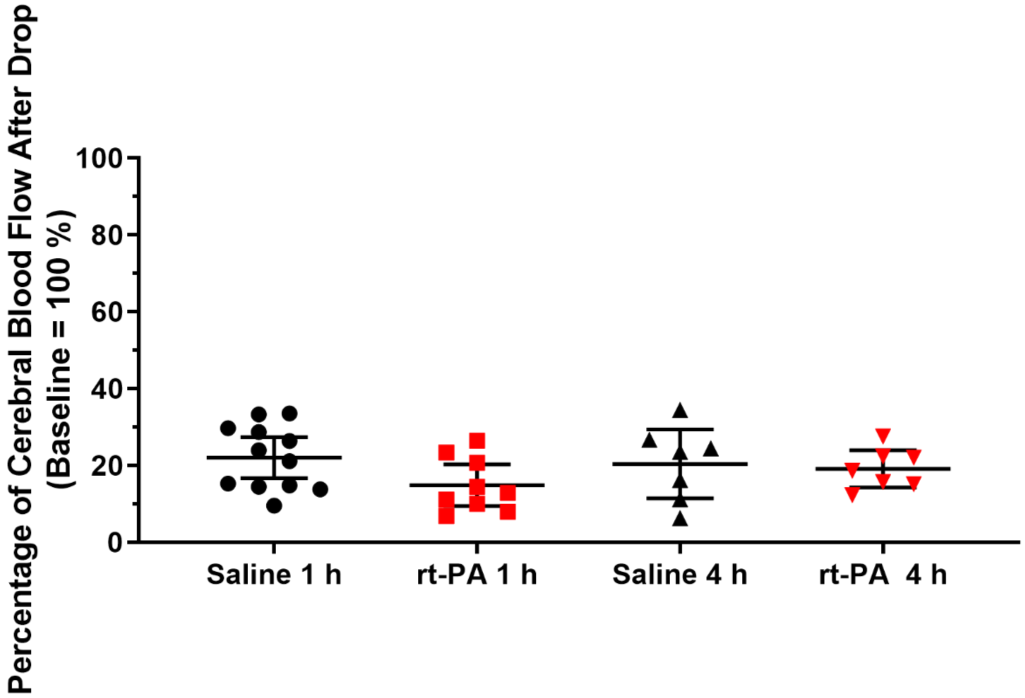

Figure S1 (a) There was no difference in weight between the different treatment groups. **(b)** Cerebral blood flow (CBF)was monitored in the middle cerebral area (MCA) during surgery using a laser Doppler. After thrombin a rapid drop of CBF was seen in the MCA, and difference in drop was seen between the different treatment groups. Data are expressed as mean and error bars as 95 % IC.

Figure S2

a

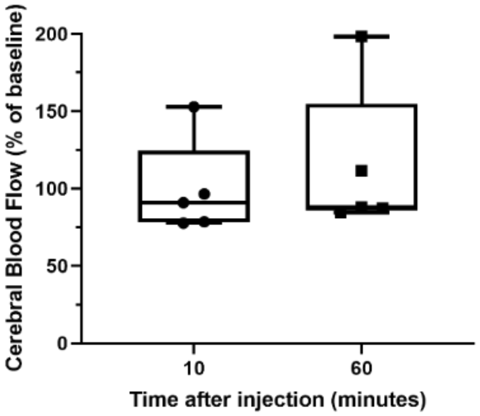

b

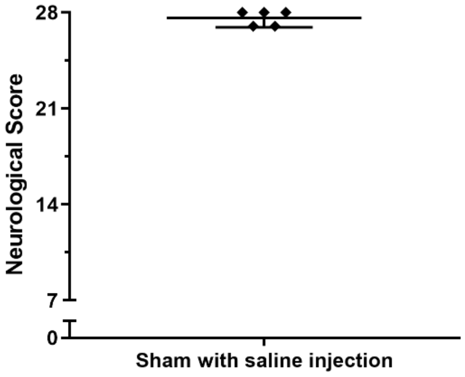

c

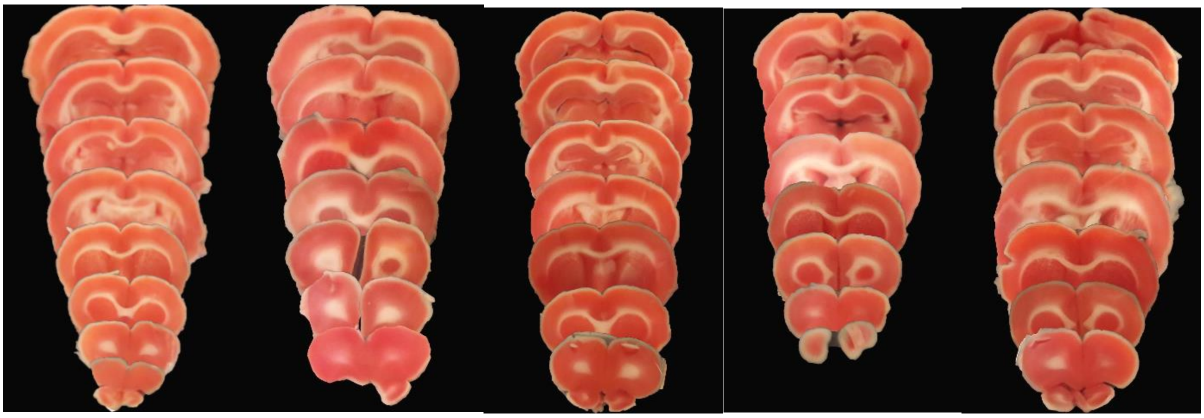

d

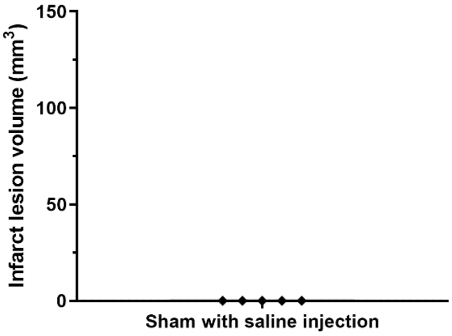

Figure S2. Sham operated rats with saline injection into the MCA. a) Sham operated animals showed no indication of drop in CBF after saline injection and CBF remained stable 60 min after injection, box-plot with minimum to maximum with mean. b) Neurological function remained normal after sham operation. c) Image of TTC-stained sham operated rats 24 h after saline injection. d) No infarct lesion formation was visualized.

Figure S3

a

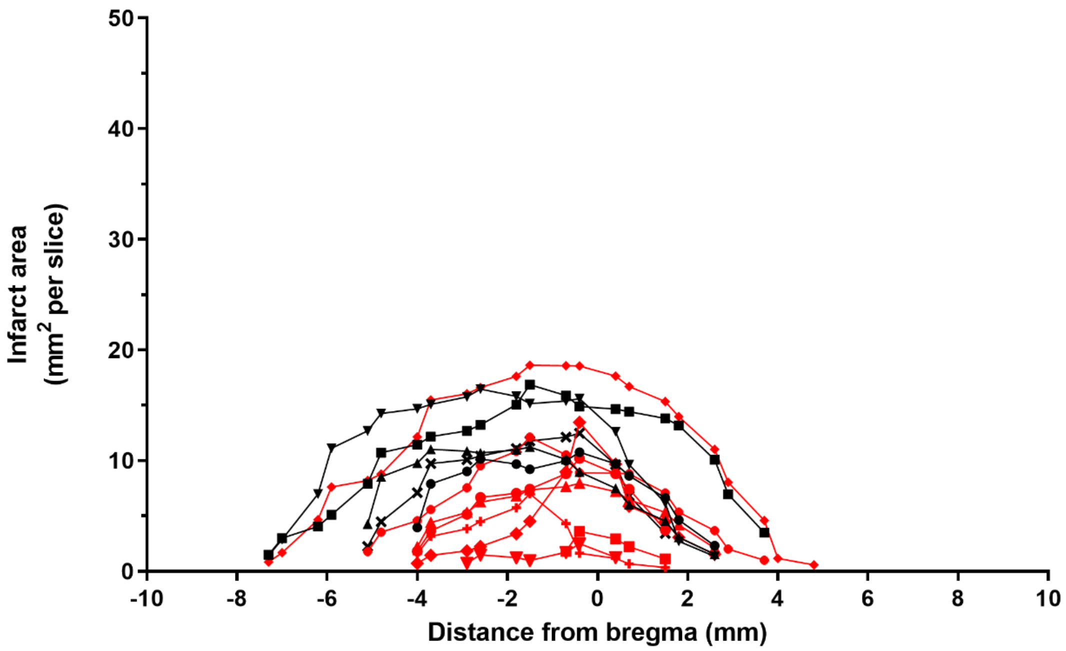

b

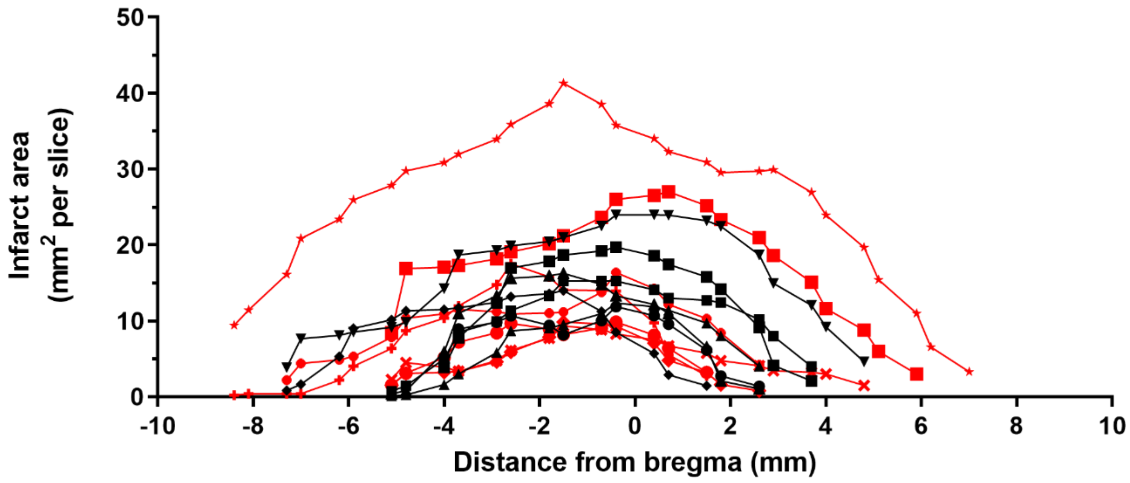

Figure S3 Infarct lesion is visualized as area of lesion per slice for each individual animal treated at 1h **(a)**, and 4h **(b)**. Saline treated animals are visualized in black, while red visualize the infarct lesion area for rt-PA treated animals.

Figure S4

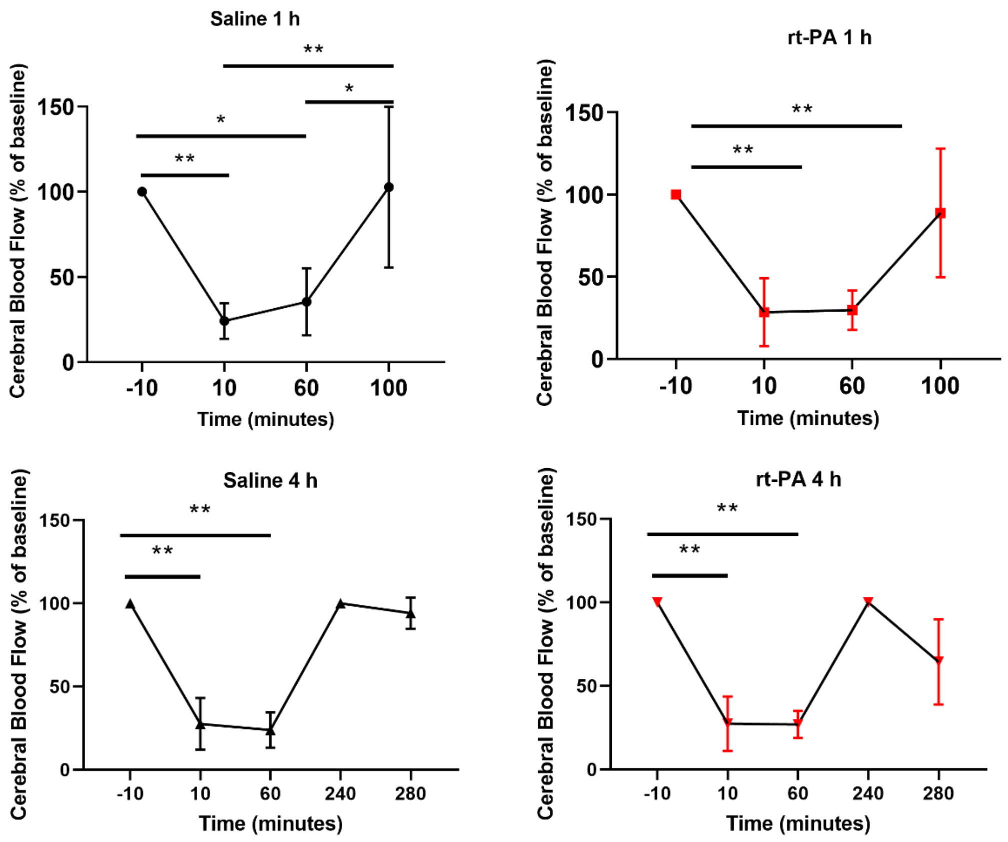

Figure S4 CBF was measured in the MCA region throughout the surgery and changes of the CBF is here visualized for each treatment group. Data are expressed as mean and error bars as 95 % IC.
